# Necroptosis in ALS: a hot topic in-progress

**DOI:** 10.1038/s41420-021-00458-4

**Published:** 2021-04-14

**Authors:** Mathilde Chevin, Guillaume Sébire

**Affiliations:** Department of Pediatrics, McGill University, Research Institute of the McGill University Health Centre, 1001 Decarie Boulevard, Montreal, QC H4A 3J1 Canada

**Keywords:** Amyotrophic lateral sclerosis, Necroptosis

**Fig. 1:**
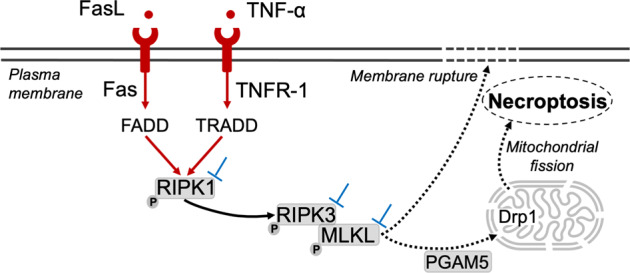
Schematic overview of necroptotic cell death. Necroptosis is triggered by inflammatory mediators, including TNF-α and FasL. Upon the recruitment of adapter proteins, the phosphorylation of RIPK1 is induced. The necrosome is composed of interaction and activation (i.e. phosphorylation) of RIPK1, RIPK3, and MLKL. Necroptosis of the cell is induced by mitochondrial fission through interaction of MLKL with mitochondrial phosphatase PGAM5 and Drp1 recruitment. Phosphorylated MLKL also triggers cell death through the disruption of the plasma membrane integrity^1,2^. Other mechanisms by which MLKL induces cell death remains to be elucidated. Pharmacological or genetic modulations (⊥) of necroptosis signaling can target RIPK1, RIPK3, MLKL, as well as their phosphorylated forms^12–14^. Drp1 dynamin-related protein 1, FADD Fas-associated protein with Death Domain, Fas-L Fas-ligand, MLKL mixed lineage kinases domain-like, p phosphorylated, PGAM5 phosphoglycerate mutase family member 5, RIPK receptor-interacting protein kinases, TNFα tumor necrosis factor α, TNFR1 tumor necrosis factor receptor 1, TRADD tumor necrosis factor receptor type 1-associated death.

Interest in potential implications of necroptosis (Fig. 1)—i.e., a recently uncovered programmed cell death pathway—in neurodegenerative diseases has been growing over the recent years^1,2^. However, very few studies addressed its role in amyotrophic lateral sclerosis (ALS). Dominguez et al.^3^ recently brought some negative findings on this topic: (i) the genetic inactivation of receptor-interacting protein kinase (RIPK)1 did not protect against motor neuron degeneration in the superoxide dismutase (SOD)1 model of ALS and (ii) phosphorylated (p)-RIPK1 did not accumulate in the spinal cords of ALS compared to non-ALS patients. These findings raise doubts about the implication of necroptosis in the pathophysiology of ALS. In contrast, other studies detected robust activation of RIPK1, RIPK3, and mixed lineage kinases domain-like (MLKL) proteins in preclinical as well as clinical studies of ALS^4–7^. The discrepancies between these studies^4,5^ and others^3,8^ might be explained by (i) the use of western blotting in whole-tissue extracts^4,5,8^ versus in situ immunolabelling targeting motor neurons^7^, (ii) the removal of circulating blood cells—a rich source of necroptotic markers^9^—in some studies but not in others, (iii) the various time frame of tissue sampling, which matches or not the transient expression of necroptotic markers^10^, or (iv) technical limitations in the availability and specificity of in vivo tools of detection of necroptotic markers^3–5,7,8^.

Hence, the involvement of necroptosis in the pathophysiology of ALS remains questionable. However, the studies showing the activation of the necroptotic pathway are based on consistent results assessing all molecular steps of this cascade namely RIPK1, p-RIPK1, RIPK3, MLKL, p-MLKL, or p-RIPK3, and tumor necrosis factor (TNF)-α^4,7^, including in situ experiments focusing selectively on the cells of upmost interest, i.e. motor neurons of the anterior horn of the spinal cord^7^. In contrast, the studies showing the absence of activation of necroptosis assessed only one molecular step of the cascade (p-RIPK1 or unphosphorylated RIPK1)^3,5^ were often untargeted on specific neural cells, and not always targeting the phosphorylation of key necroptotic markers, which are fundamental to demonstrate the execution process of necroptosis (Fig. 1)^11^.

Altogether, the final conclusion about the role of necroptosis in ALS and the interplay between the various molecular mediators and cell subtypes involved would deserve further descriptive clinical findings on larger cohorts of ALS patients, as well as preclinical studies. Such research should combine pharmacological or genetic modulations of necroptotic signaling in ALS models to uncover the pathophysiological mechanisms potentially at play in the occurrence of ALS and define the key check points of putative intervention^12–14^.
